# Gun-Shot Wound of Lung, Liver and Intestines

**Published:** 1864-03

**Authors:** R. W. Jeffery

**Affiliations:** Surgeon C. S. N., in charge Naval Hospital, Savannah.


**Art. VI.**—*Gun-shot Wound of Lung, Liver and Intestines.* Reported by R. W. Jeffery, Surgeon C. S. N., in charge Naval Hospital, Savannah.

Frank McCann, æt. 24, born in Ireland, seaman attached to C. S. Steamer Isondiga, Savannah, Georgia, at a brothel on the 1st instant, about half-past twelve o'clock at night, received a gun-shot wound in the chest. He had been drinking quite freely before the reception of the wound. The person who shot him was above McCann, on the upper part of the stairway, McC. being near the last step, and appears to have been turning, for the purpose of leaving, when he was shot.

The assistant surgeon, who saw him soon after he was wounded, not being able to trace the ball, had him brought to the hospital and gave him half grain sulphate morphia.

I saw him, for the first time, about 6 o'clock in the morning. He was then lying on the back, expression of face haggard; skin cold and clammy; respiration hurried and laboured; lips livid; pulse small, quick, and very frequent; thirst intense; vomited continually after drinking water; complained of pain in region of bladder, which he ascribed to retention, saying that he "could not pass his water;" introduced catheter, only a few drops of clear urine passed, but still he suffered from urinary irritation, which was relieved by the administration of an enema of 40 drops tincture of opium. On an examination of the wound, discovered that the ball had passed into the cavity of the chest obliquely from the neighborhood of the angle of the fourth rib of the right side; *extensive emphysema* around the wound, over right side of chest; there was but little external hemorrhage; no *cough nor expectoration;* ordered 1½ grs. pulverized opium, to relieve his pain, which was very great in lower portion of abdomen, and directed a bandage to chest. With the exception of the vomiting, his symptoms were relieved. About an hour after, saw him again, when it was discovered that his respiration was more hurried, his pulse increased in frequency and very weak; vomiting continued, and he was lying on his left side; asked him if he could not lie on the right, said "no, he could not breathe if he did;" had him raised in bed and examined his lung—respiratory murmur in right side not to be heard; percussion gave a dull sound. From this time he declined rapidly, and died at half-past three o'clock, P. M., or ten hours after the reception of his wound. His intellect was clear up to a few moments of his death.

*P. M. Examination.*—Face and body much discolored from congestion, in large blotches of a very deep crimson hue, and chest much enlarged by emphysema; ball had entered about one and a half or two inches below the wound, into the cavity of the chest, passed through the lower portion of the lower lobe of the right lung, through the diaphragm into the right portion of the right lobe of the liver; thence into the small intestines, which were perforated several times, and passed into the muscles of the iliac region of the left side, in which it was imbedded. Ball was removed and found to be a slug apparently of a Colt's revolver. The right lung was crepitous; in pleural cavities of both sides were serum and blood; in peritoneal cavity there was also bloody effusion; on omentum was discovered, in two places, coagulated blood; anterior and interior surface of stomach much injected.

On opening the abdominal cavity, an escape of gas or air issued, which might have come from the lungs, through the perforated diaphragm, or from the perforations in the small intestines—more probably the latter from its odor.

This case derives its interest from the great extent of injury and from the importance of the parts injured; lung, liver and small intestines. Some of the most common symptoms of injury of the lung were absent, I mean bloody expectoration and cough. Another equally characteristic of the injury, though less common, presented itself, emphysema. The frequent rigors, great thirst and constant vomiting, indicated the injury done to the bowels, though the thirst would also indicate great loss of blood, and might also have been caused by the debauch of the previous night; irritation of the bladder, caused probably by effusion of blood on the peritoneal covering of that viscus. Since the beginning of this war this is the second case of wounded lung in which, if my memory serves me right, I have seen emphysema, and in which there was no expectoration of blood.

